# Flagella at the Host-Microbe Interface: Key Functions Intersect With Redundant Responses

**DOI:** 10.3389/fimmu.2022.828758

**Published:** 2022-03-24

**Authors:** Douglas T. Akahoshi, Charles L. Bevins

**Affiliations:** Department of Microbiology and Immunology, School of Medicine, University of California, Davis, Davis, CA, United States

**Keywords:** fliC, goblet cell, IBD, DEFA6, LYPD8, ZG16, TLR5, IgA

## Abstract

Many bacteria and other microbes achieve locomotion *via* flagella, which are organelles that function as a swimming motor. Depending on the environment, flagellar motility can serve a variety of beneficial functions and confer a fitness advantage. For example, within a mammalian host, flagellar motility can provide bacteria the ability to resist clearance by flow, facilitate access to host epithelial cells, and enable travel to nutrient niches. From the host’s perspective, the mobility that flagella impart to bacteria can be associated with harmful activities that can disrupt homeostasis, such as invasion of epithelial cells, translocation across epithelial barriers, and biofilm formation, which ultimately can decrease a host’s reproductive fitness from a perspective of natural selection. Thus, over an evolutionary timescale, the host developed a repertoire of innate and adaptive immune countermeasures that target and mitigate this microbial threat. These countermeasures are wide-ranging and include structural components of the mucosa that maintain spatial segregation of bacteria from the epithelium, mechanisms of molecular recognition and inducible responses to flagellin, and secreted effector molecules of the innate and adaptive immune systems that directly inhibit flagellar motility. While much of our understanding of the dynamics of host-microbe interaction regarding flagella is derived from studies of enteric bacterial pathogens where flagella are a recognized virulence factor, newer studies have delved into host interaction with flagellated members of the commensal microbiota during homeostasis. Even though many aspects of flagellar motility may seem innocuous, the host’s redundant efforts to stop bacteria in their tracks highlights the importance of this host-microbe interaction.

## 1 Introduction

Microbes inhabit nearly every environment on Earth—ubiquity made possible by their ability to evolve and adapt to widely diverse conditions. One striking adaptation is the ability to move within a given environment, whether that be a pond, the surface of a damp leaf, or inside the human gut (1, 2). Locomotion provides the microbe advantages including the ability to seek nutrient-rich niches and acquire symbionts, as well as the ability to avoid noxious environments (1, 3–7). Many microbes achieve locomotion *via* flagella, which are “tail-like” organelles that function as a swimming motor (8, 9). The structure and biomechanics of microbial flagella have been extensively studied and reviewed (8, 10–18).

Among microbial flagella, the bacterial flagella have been the most extensively studied. Despite taxonomic diversity within the domain Bacteria, the structure and sequence of bacterial flagella are highly conserved (10). The prototypical flagellum of bacteria is comprised of three protein-based components: basal body, the hook, and filament (8, 19). The basal body is imbedded in the membrane, serving as an anchor for the hook and filament. The hook is attached to the basal body and together they generate the torque necessary to rotate the filament (8, 20). The main body of a flagellum is the filament, a multimeric polymer composed of between 100-20,000 protein monomers, termed flagellin (21). Working together, the flagellum apparatus provides bacteria the ability to move in liquid and semi-solid environments in a directional manner, reaching recorded velocities of 30µm/second (8). Commonly, this flagella-driven movement of bacteria is directed toward beneficial chemical gradients or away from toxic chemical gradients in a process termed chemotaxis (22, 23). Although the synthesis and use of flagella incurs a resource cost for bacteria, this energy debt is outweighed by the ability to gain access to throughout the environment and outcompete non-motile microbes, especially when resources are scarce (23–26).

The gastrointestinal (GI) tract of vertebrates is home to a phenomenally diverse microbial community. In this environment, some microbes incorporate flagella into their lifestyles in order to gain an advantage over their competitors. Indeed, it is well established that flagellar motility is an essential virulence factor for numerous enteric pathogens (27–31). However, flagellated microbes in the GI tract face a challenge absent in many other environments—a host that is actively sensing and restricting flagellar motility. From the host’s perspective, the mobility that flagella impart to microbes carries potential risk. For example, flagella-driven motility can facilitate cellular invasion and translocation across the epithelium. Thus, in response to flagellar activity, the host has developed a repertoire of immune countermeasures to mitigate this threat. This review will provide a perspective on various ways that animal-associated microbes utilize flagellar motility, and highlight the redundant strategies co-evolved by the host to recognize, adapt, and respond to this molecular process. Based on the prominent use of bacterial models in current literature on the topic, the focus will be on the bacterial flagella.

## 2 Flagellar Motility as a Colonization Factor

For microbes associated with the GI tract, much of our understanding of flagella is derived from the study of pathogens, since flagellar motility is often key in their successful colonization of the host. Although many different pathogens utilize flagellar motility within the host, it often provides varying degrees of fitness advantage and is not always necessary for host colonization. For example, the use of flagellar motility is integral to the pathogenesis strategy of *Helicobacter pylori* and *Campylobacter jejuni*, as evidenced by its absence hindering their ability to effectively colonize the host (1, 27, 32–35). By comparison, *Salmonella enterica* serovar Typhimurium (STM), *Escherichia coli*, and *Pseudomonas aeruginosa* selectively express their flagella at specific times and sites within the host to gain advantage over other members of the microbiota; however, these bacteria ultimately do not require flagellar motility for pathogenesis, and when expressed at the “wrong” time can actually impair colonization of the host (1, 27, 36–42). For *Listeria monocytogenes*, flagellar motility appears to play an important role outside of the host where it might increase infectious potential (43). However, upon transitioning from the cooler external environment to the warmer environment inside the host, Listeria actively downregulates flagella expression and utilizes alternative motility mechanisms (44). In addition to their fundamental role in motility, flagella can have additional functions for some bacteria, including secretion, adhesion, and biofilm formation (23, 29, 45–50). For example, a recent investigation demonstrated that STM can methylate lysine residues on its flagellin to increase the hydrophobicity of the flagella and thereby facilitate adhesion to the hydrophobic surface of host cells (48).

**Figure 1:**
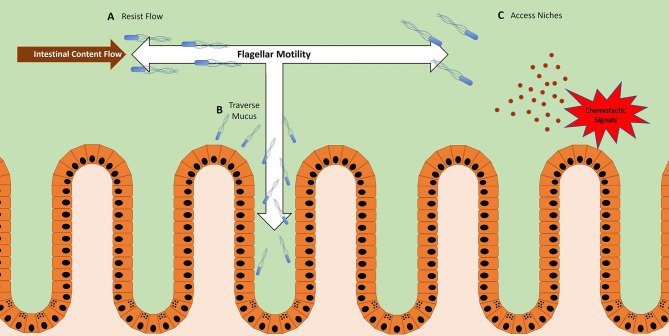
Bacteria utilize flagellar motility for multiple functions within the host. **(A)** Bacteria use flagellar motility to resist the flow of intestinal contents, maintaining their location within the host GI tract. In the case of *Vibrio cholera*, this allows them to maintain stable colonization of the proximal end of the *Danio rerio* (zebrafish) gut (51, 52). **(B)** Bacteria use flagellar motility to swim through mucus to reach the epithelium. This is a likely method for microbes that need to reach the epithelium in order to facilitate activities such as adhesion, invasion, and translocation (37, 53). **(C)** Bacteria use flagellar motility to swim toward chemotactic signals (36, 37, 54, 55). These signals can be products of inflammation or epithelial damage, representing vulnerable areas of the mucosal barrier.

Altogether, these examples illustrate how flagella expression within the host can have either positive or negative impacts on bacterial competitive fitness. To elaborate on how flagellar motility can be important to microbes within the host, three partially overlapping functions will be discussed: the power to resist flow, the ability to reach the epithelium, and the capacity to travel to nutrient niches within the lumen (**Figure 1**). 

### 2.1 Flagellar Motility, a Mechanism to Resist Flow

Given that microbes often optimize their metabolism to specific nutrient niches, it is important for these microbes to localize to the favorable regions of the host gastrointestinal tract (56). However, the proximal-distal peristaltic flow of intestinal contents, as well as the rapid turnover and shedding of mucus and epithelial cells, impose a perpetual existential threat to microbes attempting to maintain colonization (51, 52, 57). To overcome these challenges, some commensal and non-commensal bacteria use flagellar motility to stably colonize specific regions of the GI tract (57, 58).

A recent study using intravital microscopy of *Danio rerio* (zebrafish) revealed a role of flagellar motility in resisting the peristaltic flow of luminal contents in the GI tract (52). The zebrafish is an attractive model for viewing microbial motility *in situ* because during its embryonic stage the fish are transparent, allowing for live imaging of the GI tract (59–61). Using this approach, a motile strain of *Vibrio cholerae* was shown to stably colonize the zebrafish GI tract and localize to the most proximal regions of the foregut (52). However, when flagellar function was impeded, the *V. cholerae* strain was more susceptible to expulsion, thereby diminishing colonization – the remaining bacteria were observed in more distal regions of the GI tract (52). If chemotaxis rather than flagellar motility were inhibited, a similar scenario emerged but with nuanced variation. When chemotaxis was inhibited, *V. cholera* colonized efficiently but localized more distally than their WT counterparts, with a smaller fraction remaining in the proximal region of the foregut (52). Generally, inhibition of chemotaxis does not impair motility per se, but rather leaves bacteria unable to change directions in response to chemotactic signals within the environment. Without the ability to bias movement toward specific locations, bacteria tend to more uniformly disperse. In the zebrafish gut, this resulted in the dispersal throughout the lumen of chemotaxis-deficient *V. cholera*, while WT *V. cholera* concentrate closer to the epithelium. The localization closer to the epithelium likely provided protection from the flow of intestinal contents, and thus afforded WT *V. cholera* the ability to maintain stable colonization of the proximal foregut (51). Together, this study elegantly highlights how flagellar motility provides *V. cholerae* the means to resist the peristaltic flow in the intestinal tract (**Figure 1A**).

While these studies were done in zebrafish, it is likely bacteria utilize flagellar motility to similar effect within the mammalian gut. Indeed, studies on IgA agglutination of bacteria in the mouse intestine showed that agglutinated bacteria were expelled from the host (62). The proposed mechanism was that agglutination restricted motility of these bacteria, rendering them unable to resist the proximal-distal flow of intestinal contents (62). Thus, flagellar motility appears to be a viable strategy for bacteria to maintain their regional localization within the gastrointestinal tract. 

### 2.2 Flagellar Motility, a Mechanism to Acquire Proximity

During homeostasis, the GI epithelium establishes a mucus barrier that effectively segregates the luminal microbes away from the epithelial layer (63–65). In simplest terms, mucus is a macromolecular complex that forms a semi-permeable barrier adjacent to the epithelium (63). For many enteric pathogens, an aspect of their lifestyle necessitates contact with the epithelium to facilitate activities such as adhesion, invasion, and/or translocation. Thus, these microbes require a mechanism to efficiently overcome the mucus barrier.

Some enteric pathogens employ flagellar motility to traverse the mucus barrier (**Figure 1B**). *In vivo* evidence using STM models at early timepoints of infection demonstrate that flagellated bacteria are more closely associated with the epithelium than their aflagellate counterparts (37). While these data suggest the importance of flagella to access the epithelium, a caveat worth noting is that the phenotype might not be solely attributable to the loss of flagellar motility per se, since flagella can perform other functions. More direct data comes from recent work utilizing live-fluorescence microscopy of intestinal explants, which allowed the real-time viewing of individual swimming bacteria interfacing with the mucus barrier (53). While a majority of STM were observed segregated to the outer mucus layer, a significant fraction of flagellated STM successfully swam through the inner mucus layer and associated more closely to the epithelium (53). By contrast, aflagellated STM were almost completely confined to the surface of the outer mucus layer (53). These observations provide visual confirmation that STM can use flagella to swim through mucus towards the epithelium, an important property for this multi-functional organelle.

The real-time visualization of live bacteria in *ex vivo* systems also enables analysis of more subtle swimming behaviors. For example, STM swimming close to the epithelium engage in a phenomenon termed “near-surface swimming” - when STM reach an impassable surface, such as the surface of the epithelium or dense mucus layer, they initiate a circular swimming pattern (53, 66). Although a detailed understanding of this behavior is not clear, an attractive hypothesis is that near-surface swimming is a strategy to find vulnerabilities along a dense mucus layer, or identify potential attachment and invasion sites on an epithelial monolayer (66).

Surprisingly, STM lacking flagella are able to effectively colonize the host and disseminate to peripheral tissues, despite their lack of motility. This raises the question of how these bacteria reach the epithelium, despite an apparent inability to passively diffuse through mucus. A likely explanation stems from experimental observations of bacteria, irrespective of flagellar motility, coming in direct contact with epithelial cells at vulnerable sites, such as the surface-exposed Peyer’s patches (53). In addition, surface migration that does not involve flagella has been described (67). Such contact and migration could reconcile how aflagellated STM are able to infect the host, albeit less efficiently than their motile counterparts (53). The findings with aflagellated STM provide precedence for possible mechanisms for how other bacteria without a functional flagellum, such as *Citrobacter rodentium*, are capable of attaching and effacing to epithelial cells in the GI tract (68).

### 2.3 Flagellar Motility, a Mechanism to Access Nutrient Niches

The distinct environments of various regions within the GI tract create niches that accommodate microbes with an array of lifestyles (56). Moreover, within each individual niche there are microniches, owing to factors such as the topography and inflammatory state of the epithelium (56, 69, 70). Thus, microbes possessing the necessary metabolic machinery to exploit these microniches can gain a significant advantage over their competitors — as long as they have the ability to detect and physically access these sites (25, 26). For some bacteria, this ability comes in the form of chemotaxis-directed flagellar motility (**Figure 1C**).

In the stomach, *H. pylori* requires chemotaxis to occupy microniches within the gastric gland, which confers a competitive edge over other strains (54, 71). In response to gastric ulcers, some *H. pylori* strains sense chemotactic signals associated with epithelial damage and travel to those sites *via* flagellum-powered migration (55, 72). *H. pylori* presence at the ulcer sites delay healing, thereby maintaining the microniche and benefiting from the associated nutrients (55).

Similarly, STM employs chemotaxis in the distal intestinal tract to gain a competitive advantage in the inflamed colon (36, 37). Specific methyl-accepting chemotaxis proteins are expressed by STM, allowing them sense and swim towards nitrogen-rich nutrient niches (36) (**Figure 1C**). These niches are often generated as byproducts of the inflammatory response of the host, likely derived from the reactive oxygen and reactive nitrogen species secreted by the epithelium (36). According to proposed models, the inflammatory response induced by STM leads to the creation of these nutrient niches, which attracts STM migration *via* flagellum-driven chemotaxis (36). Thus, through their use of flagellum-driven chemotaxis, both *H. pylori* and STM illustrate how the flagellum can be used as a tool to reach specific nutrient microniches within the host and secure a competitive advantage over other microbes. Interestingly, a recent study demonstrated that “minicells”, a term coined for chromosome-free nanosized bacterial vesicles, could be engineered to express flagella and bias their movement towards chemical gradients, which resulted in their accumulation at the chemoattractant source (73). 

## 3 A Host’s Perspective to Flagella-Driven Motility

In isolation, microbes swimming in the lumen *via* flagella-driven motility would not appear to cause harm or pose a threat to the host. However, as noted, some microbes use this flagellar motility as a step towards epithelial adherence and translocation, a process detrimental to host fitness. To mitigate this threat, the host expends considerable resources to maintain an arsenal of often redundant countermeasures to impair flagella-driven motility (74, 75). For example, the importance of the host’s response to flagellated microbes is evident in mice deficient in either TLR5 or NLRC4, which because of their inability to effectively detect flagellin are more susceptible to basal inflammation and infection by enteric pathogens (76–81). Furthermore, adaptive immunity, and in particular mucosal production of flagellin-specific IgA, is also of vital importance in keeping motile bacteria in check (82). The next two sections will delve into the mechanisms that the host uses to hinder flagella-driven motility, including both innate and adaptive immune responses.

### 3.1 Innate Immune Countermeasures

Innate immunity is marked by providing the host with an ever-ready or immediately inducible collection of molecules that can effectively mitigate microbial threats and restore homeostasis. With respect to innate immune molecules and responses that may counter the possible deleterious consequences of flagella-driven motility, five mechanisms have been identified and highlighted here.

#### 3.1.1 Mucus Barrier

Mucus plays an essential role in maintaining a stable homeostasis between the host and microbes, in part, acting as a physical barrier to fortify the epithelium (63–65, 83). The effective barrier properties of mucus are especially evident in the colon, where the extraordinary density of resident microbes are partitioned away from the epithelial surface. Based on FITC-dextran permeability assays, the dense inner layer of colonic mucus immediately adjacent to the epithelium is selectively permissive to particles smaller than 0.5 µm in size. While the shapes and dimensions of microbes vary, many bacteria fall in the range of 0.5-1.0 µm in length; thus, they would not passively diffuse through the mucus barrier due to Brownian motion. Accordingly, experiments utilizing fluorescence *in situ* hybridization (FISH) to label bacteria show that the vast majority of the microbes reside either in the lumen or penetrate only to the more porous outer mucus layer, suggesting an inability to pass into the inner mucus layer adjacent to the epithelium. Yet, some microbes can be observed swimming through the inner mucus and able to reach the epithelium, most likely utilizing flagellar motility (53).

While flagellar motility allows microbes to penetrate the mucus layer, a percentage of these bacteria become “entrapped” in the mucus and are immobilized (53). Somewhat counterintuitively, entrapment within the mucus layer appears to be not dependent on the expression of flagella per se, but rather on the locomotion imparted by flagellar motility (53). As such, bacteria possessing a functionally disabled flagellum do not become entrapped within the mucus layer. The exact mechanism that enables mucus to target motility of actively swimming microbes for entrapment remains unclear. In addition to this intrinsic ability to hinder motility, mucus also harbors an array of host derived immune factors that reinforce barrier function (84) (**Table 1**). These will be addressed in some detail. 

**Table 1:** Innate immune effector molecules.

Effector Molecule	Proposed Mechanism	References
Mucus	Barrier	(53, 63–65, 83–85)
	Scaffold for Other Effectors	
	Bulk Flow from Crypts	
HD6	Agglutination	(86–88)
	Inhibit Flagella Activity ()?	
LYPD8	Inhibit Flagella Activity	(89–91)
ZG16	Agglutination	(92, 93)
	Redox Switch	

#### 3.1.2 Flagellar-Binding Proteins and Peptides

**Figure 2:**
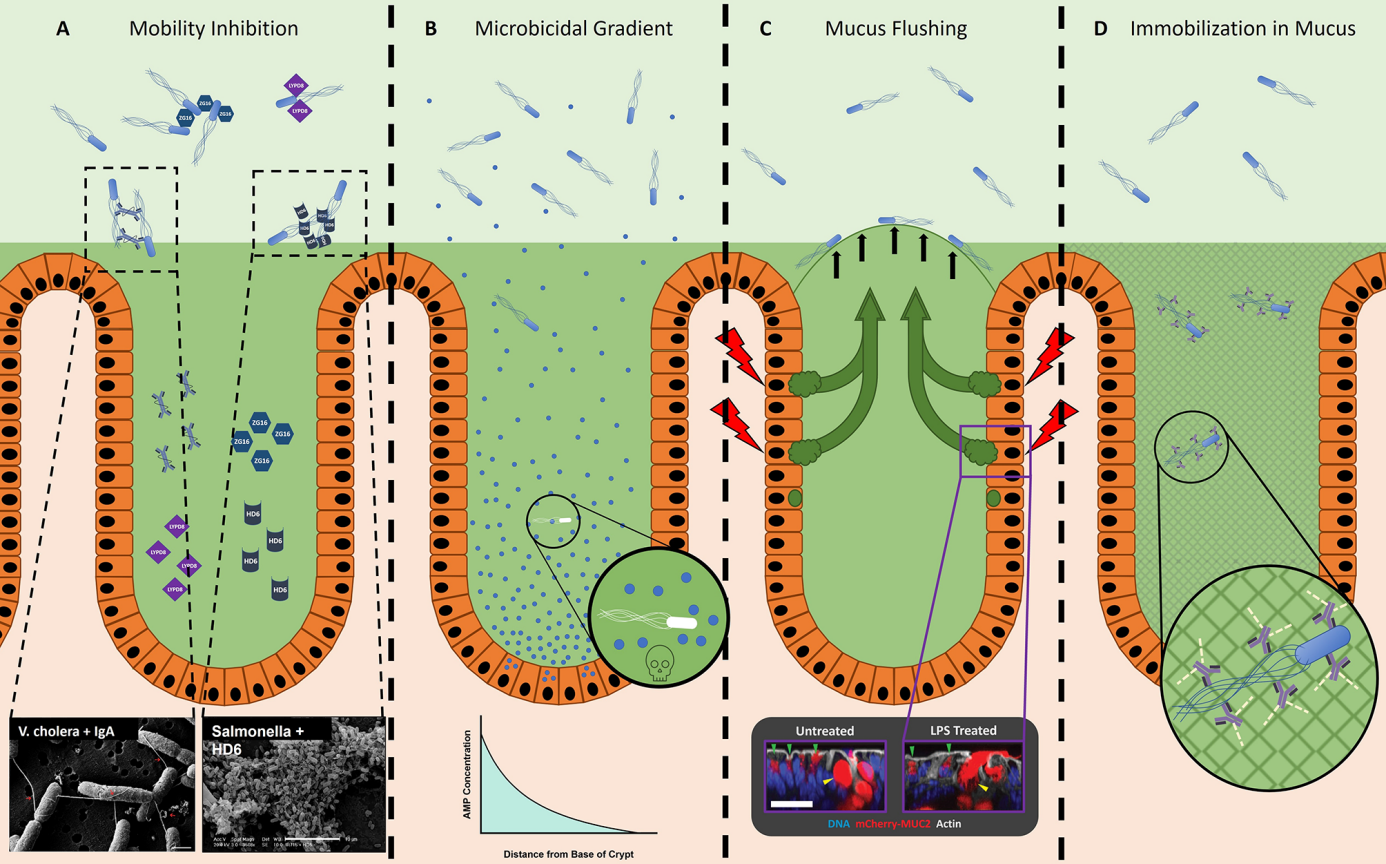
The host prevents microbes from accessing the epithelium. **(A)** Innate effector molecules and antibody are present in the GI tract lumen and inhibit bacterial motility through various means. ZG16 (blue hexagons) aggregates motile Gram-positive microbes in the outer mucus layer, away from the epithelium (92). Lypd8 (purple squares) inhibits bacterial motility in the colon through a currently unknown, non-agglutinating mechanism (89). Dotted boxes depict examples of SEM micrographs of sIgA (Left) and HD6 (Right) agglutinating bacteria, taken from Levinson et al., 2015 (62, 94) and Chu et al., 2012 (86), respectively. HD6, human α-defensin 6; LYPD8, Ly6/plaur domain containing 8; ZG16, zymogen granular protein 16. **(B)** Microbicidal effector molecules in the small intestine form a concentration gradient, with the highest concentration located in the crypt (95). It is likely that microbes able to localize closer to the crypts will be subjected to a more inhospitable or fatal environment, as depicted in the magnified image. AMP, Antimicrobial Peptide. **(C)** Sentinel goblet cells specialized goblet cells located at the entrance of colonic crypts (85). After stimulation with a bacterial ligand such as flagellin or LPS (red lightning bolt), these sentinel goblet cells will secrete mucus into the crypt. This is likely a method to detect microbes that have traveled through the inner mucus layer (possible by flagellar motility) and initiate the secretion of extra mucus (green arrows), thereby effectively flushing the microbes away from the crypt (black arrows). Dotted box depicts a sentinel goblet cell secreting mucus (right) in response to stimulation by a bacterial ligand, compared to an unstimulated sentinel goblet cell (left), taken from Birchenough et al., 2016 (85). **(D)** IgG-bound bacteria become immobilized in mucus due to the collective minor and non-covalent interactions (green dashed lines) between IgG and mucus (grey mesh) (96–98).

Various innate immune proteins bolster the efficacy of the mucosal barrier against noxious microbes, and some are embedded in mucus (84). These effector molecules include peptides and proteins secreted by the epithelium. While many of these molecules are microbicidal, a subset provide protection through alternative mechanisms such as inhibition of motility and spatial segregation. Included in this latter group are human α-defensin 6 (DEFA6, HD6), Ly6/Plaur domain containing 8 (LYPD8), and zymogen granular protein 16 (ZG16), all of which appear to affect the motility and localization of bacteria, although by different, non-bactericidal mechanisms (**Figure 2A**).

##### 3.1.2.1 HD6

HD6 is produced by Paneth cells in the crypts of the small intestine (86, 99, 100). Unlike most processed and folded α-defensins that are potently bactericidal, HD6 lacks antimicrobial activity (86). Recent studies have reported antimicrobial activity of proteolytic fragments of HD6 (101), and activity against anaerobic commensal bacteria for HD6 when its disulfide bonds are reduced (102). Instead of microbicidal activity, processed and folded HD6 binds proteins on bacterial surfaces and polymerizes to form macromolecular fibrils and “nets”, which can agglutinate bacteria into immobilized aggregates (86–88). During STM infection, the presence of HD6 in transgenic knock-in mice significantly limited invasion and dissemination of STM to peripheral tissues, without decreasing the bacterial density in the intestinal lumen (86). Consequently, these mice were more able to survive the infection compared to their WT counterparts (86). Interestingly, HD6 appears to selectively target non-glycosylated proteins; this is significant since bacterial surface proteins, which include flagellin, are not typically glycosylated (86), although exceptions exist (34, 103–105). Thus, HD6 can provide the host a mechanism to immobilize flagellated bacteria, such as STM. Orthologs of HD6 are reported in Rhesus macaques and some other non-human primates, but not in murids (86).

##### 3.1.2.2 LYPD8

**Figure 3:**
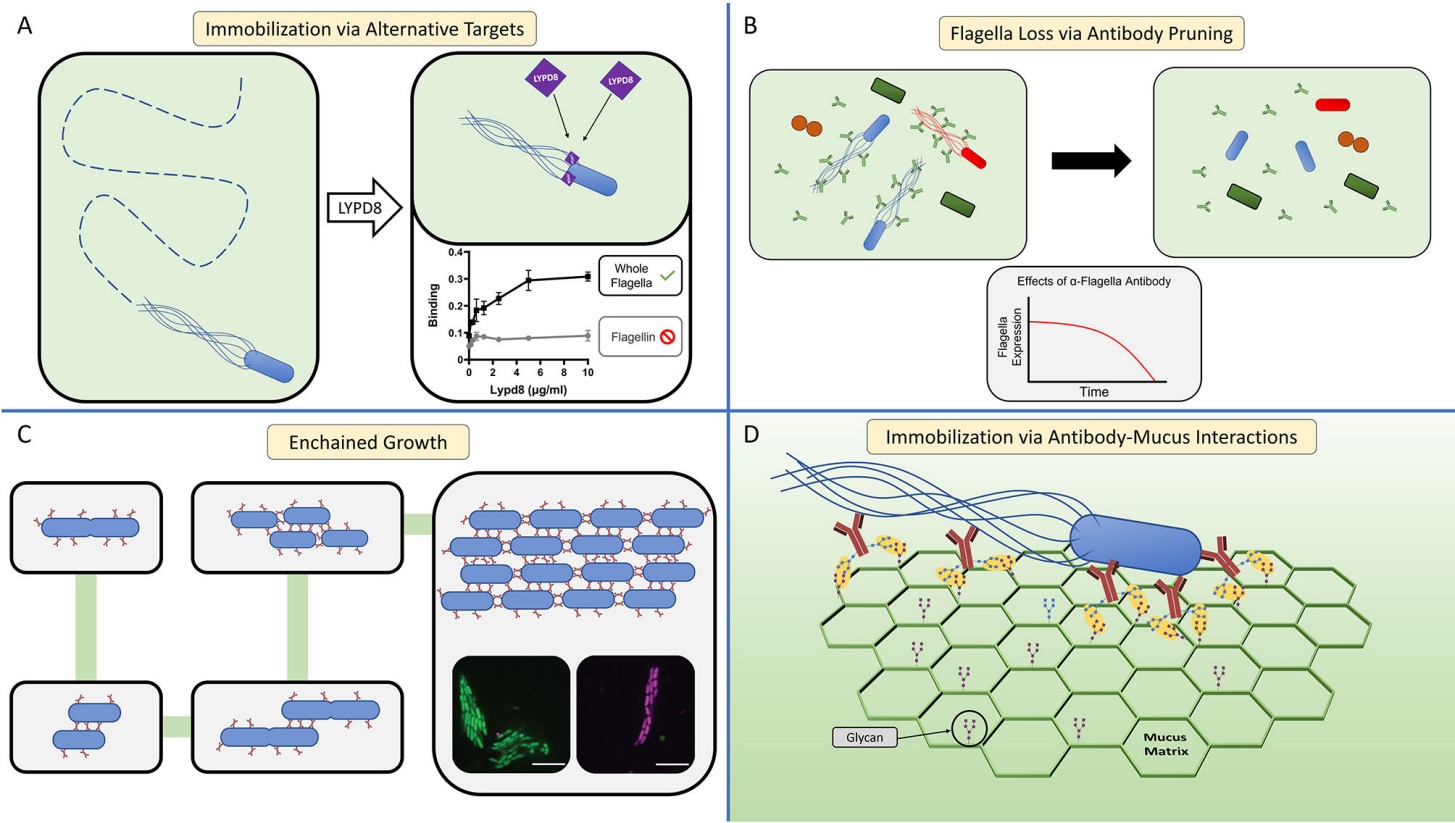
The host immune system can inhibit bacterial motility through multiple mechanisms. **(A)** Host effector molecules can inhibit flagellar motility through non-agglutination means by binding sites on the flagellum other than flagellin. LYPD8 (purple diamond) inhibits the flagellar motility of STM by binding to the flagellum (89). Graphs display ELISA experiments showing that LYPD8 binds the flagella (right), but does not specifically target flagellin (left) [adapted from (89)]. LYPD8, Ly6/Plaur Domain Containing 8. **(B)** The host can influence the microbiota’s expression of flagellar genes through antibody-mediated pruning (74, 107). A sizable fraction of antibody (green bifurcating structures) in the GI tract bind can bind to the flagella of commensal microbes (left panel), resulting in a significant decrease of flagellated bacteria within the microbiota (right panel). While this antibody-mediated pruning results in a decrease of flagella expression within the microbiota, the overall composition of the microbiota remains largely unchanged. *In vitro* experiments show that the anti-flagellin antibody causes *E. coli* to down regulate its expression of flagellin over time (graph). **(C)** In the GI tract, antibody-mediated agglutination can occur through a process termed enchained growth (62, 94). By this mechanism, bacteria that are actively dividing within the GI tract are coated in antibody (red line structures). Upon successful fission, the daughter cell is immediately linked to the mother cell from antibody crosslinking. Over multiple cycles of division, an agglutinated aggregate is formed consisting of clonal population of bacteria. Fluorescence microscopy micrographs depict examples of these clonal agglutinated aggregates (inset micrographs). **(D)** Antibody-mucin interactions facilitate the immobilization of motile bacteria within the mucus matrix (green hexagonal structure). In this model, glycans present on the surface of antibody (brown bifurcating structures) and mucin form weak, non-covalent interactions (yellow ovals) (96, 97). When motile bacteria within the mucus are coated by antibody, the numerous antibody-mucus interactions create a cumulatively strong non-covalent interaction that immobilizes the bacteria.

LYPD8 is abundant in the large intestine where it is produced by colonic epithelial cells (89). Like HD6, LYPD8 lacks discernable bactericidal activity; nevertheless, it also provides protection from pathogen challenge (89, 106). When LYPD8 is absent from the colonic mucus, bacteria localize closer to the epithelium. Additionally, *in vitro* data show that LYPD8 inhibits bacterial motility, suggesting that LYPD8 maintains the spatial segregation of bacteria and epithelium by preventing motile bacteria from swimming through the mucus layer (89–91). In support of this hypothesis, LYPD8 selectively targets flagellated bacteria; however, LYPD8 does not bind flagellin, implying it may instead bind to another component of the flagellum (**Figure 3A**), such as the hook or basal body (89). Unlike HD6, LYPD8 does not appear to agglutinate bacteria, and the molecular mechanism by which LYPD8 interferes with bacterial motility remains unclear.

##### 3.1.2.3 ZG16

ZG16 is also produced by the colonic epithelium and found in the colonic mucus (92). Similar to the effects of removing LYPD8, the absence of ZG16 also leads to bacteria localizing closer to the epithelium (92). However, in contrast to both LYPD8 and HD6, which appear to bind to protein ligands, ZG16 *i*) contains a CXXC motif on its flexible carboxy terminus that may serve a redox switch to alter its protein conformation (93), and *ii*) is a lectin that specifically binds to the peptidoglycan of Gram-positive bacteria (92). After binding, ZG16 aggregates bacteria, thereby limiting their motility (92). Through this mechanism, ZG16 likely fortifies the barrier to motile Gram-positive bacteria, which ultimately restricts their access to the colonic crypts and epithelium (92). Consequently, mice lacking ZG16 possess a higher burden of Gram-positive 16sRNA in circulation and peripheral tissues, implying that the closer proximity to the epithelium of Gram-positive bacteria leads to increased rates of translocation (92). 

#### 3.1.3 Effector Gradients

In addition to the repertoire of effector proteins that function *via* mechanisms of motility inhibition, bactericidal peptides and proteins produced in the GI tract also contribute to the maintenance of spatial segregation. At the base of small intestinal crypts, secretory Paneth cells constitutively secrete antimicrobial peptides and proteins (AMPs) into the lumen, creating a concentration gradient with highest concentrations at the base adjacent to the intestinal stem cells (95) (**Figure 2B**). Consequently, luminal bacteria entering the crypt would initially be exposed to mucus harboring a lower concentration of AMPs, and as such, subjected to less bactericidal activity; however, bacteria able to swim into the crypt would be exposed to elevated concentrations of AMPs, a more inhospitable (and potentially lethal) environment (95, 108). In support of this model, Paneth cell ablation does not result in microbial overgrowth in the lumen as one might initially expect, but instead results in increased rates of bacterial translocation likely due to a more permissive environment near the epithelium and crypt (109). Therefore, the minefield of AMPs produced by Paneth cells may provide an “incentive” to stay out of the crypt, and instead remain distant from the epithelial surface.

The importance of this Paneth cell function is highlighted in the context of chronic inflammatory bowel disease (IBD), where the morphology and function of Paneth cells are impaired (110–114). In Crohn’s disease of the small intestine, a major type of IBD, the expression of Paneth cell α-defensins is reduced, potentially related to genetic impairments in bacterial recognition and the autophagy response (110, 111). Since Paneth cell ablation is linked to increased rates of translocation, a similar scenario might occur in Crohn’s disease wherein the reduction of Paneth cell products permits microbes to swim to the small intestinal epithelium and breach the barrier to initiate inflammation (109). Thus, the higher levels of flagellin-specific antibody found in Crohn’s disease patients could be due to a luminal environment more permissive to flagellar motility and access of flagellated bacteria to the mucosa (115). Consistent with this model, the major cell type in the small intestinal epithelium expressing TLR5 is the Paneth cell (116). During homeostasis, this pairing suggests the potential for a nuanced relationship between Paneth cells and motile bacteria. However, with the inflammatory conditions characteristic of Crohn’s disease, Paneth cells are likely under stress that results in impaired function, which could be further exacerbated by excessive TLR5 signaling due to elevated number of flagella. Since Paneth cells play a role in maintaining host-microbe homeostasis, their impaired function may perpetuate the chronic inflammation that impedes a return of homeostasis.

#### 3.1.4 Sentinel Goblet Cells

Recently, a specialized subset of goblet cells, termed sentinel goblet cells, were discovered in the colonic epithelium positioned adjacent to the entrance to the crypt (85). What defines this subset of goblet cells is their ability to secrete appreciable amounts of mucus in response to pattern recognition receptor (PRR) stimulation. Notably, TLR5 is among the PRRs expressed by sentinel goblet cells, and as such, stimulation with flagellin is sufficient to induce mucus secretion. Birchenough et al. propose a model wherein sentinel goblet cells hold watch at the crypt opening; if microbes bypass the inner mucus layer and come in close proximity to the epithelium, then these cells will secrete a burst of mucus to effectively “flush” the crypt (**Figure 2C**). Since flagellar motility provides microbes the means to reach the colonic epithelium, and a ligand to stimulate TLR5, the function of sentinel goblet cells may be to protect vulnerable crypts from flagellated microbes.

### 3.2 Adaptive Response

Like the innate immune system, the adaptive immune system can effectively inhibit microbial motility, mainly through luminally secreted antibody. Although neutralization, opsonization, and complement activation are the typical mechanisms ascribed to antibody activity, alternative mechanisms are employed within the GI tract lumen to address flagellar motility during both homeostatic and inflammatory states. These mechanisms include pruning, agglutination, and immobilization.

#### 3.2.1 Antibody Pruning

During homeostasis, plasma cells in the GI tract continually secrete commensal-specific IgA and IgG into the intestinal lumen (117). While these antibodies target a number of microbe-surface antigenic sites, a sizable fraction bind specifically to bacterial flagellin (74, 118, 119). Within the diverse gut microbial community, many bacteria possess the genes necessary to produce functional flagella (74). However, meta-transcriptomic data indicate that a relatively small proportion of those bacteria are actively expressing flagellar genes in the intestine under homeostatic conditions (74).

Notably, the presence of this flagellin-specific antibody repertoire appears largely dependent on TLR5 expression, consistent with studies indicating that TLR5 selectively enhances flagellin-specific CD4 T cell responses (74). Using a TLR5 genetic knock-out mouse model, Cullender and colleagues found that TLR5 expression facilitates flagellin-specific antibody production, and in turn is inversely linked with expression of flagellar genes in commensal microbes (74). Consequently, in mice lacking TLR5, flagellated microbes are more free to swim within the mucus, which establishes new baseline conditions where flagellar motility is less restricted and the spatial segregation of microbes and epithelium is compromised. The mechanism driving the inverse relationship between flagellin-specific antibody and flagella expression was termed microbial “pruning”, wherein microbes alter their expression of surface molecules in response to being bound by antibody (**Figure 3B**) (74, 107). While the exact molecular mechanism explaining how antibody causes this effect remains unknown, observations of *E. coli* sensing mechanical stimuli through their flagella could provide a possible explanation (120–122). Thus, the host utilizes antibody as a tool to inhibit expression of flagella by colonizing microbes, thereby creating conditions of homeostasis that include the restricted motility of commensal microbes.

#### 3.2.2 Agglutination

For close to a century the process of antibody-mediated agglutination has been utilized in clinical medicine and basic research to identify the “serotype” of bacteria, such as STM and *E. coli*, based on specific bacterial-surface antigens. Simply mixing isolated bacteria with immune serum (or purified antibodies) yields visible aggregates of agglutinated bacteria. While the practical application of antibody-mediated *in vitro* agglutination is clear, our knowledge of how agglutination occurs *in vivo* is less complete.

In the GI tract, antibody-mediated agglutination was thought to occur through the same mechanism as with *in vitro* agglutination assays, the so-called classical agglutination model. This model was based on antibody-coated, planktonic bacteria colliding and sticking together, over time forming larger and larger aggregates. In this model the rate of agglutination is heavily dependent on the concentration of bacteria. However, even when *in vivo* bacterial concentrations are orders of magnitude below the predicted minimum concentration for classical agglutination to occur, antibody-mediated agglutination is still observed (62). Moor and colleagues solved this enigma by showing that when bacterial concentrations are too low for classical agglutination to occur, the active replication of antibody-coated bacteria creates the conditions allowing agglutination to occur—a process termed enchained growth (62, 94). The enchained growth model holds that during conditions where bacteria are replicating in the presence of antibody, the newly replicated daughter cells initially will be linked to their mother cells, which after a few cycles of division will result in the agglutination of an entire lineage (**Figure 3C**). While the rate of classical agglutination is a function of the initial bacterial concentration, the rate of agglutination through enchained growth is a function of the rate of bacterial division.

Once agglutinated, the bacteria are no longer motile even if possessing functional, and sometimes actively rotating, flagella (62, 94). As a consequence, agglutinated bacteria are eventually expelled from the host, likely due to the peristaltic flow of intestinal contents, in a process previously described for the zebrafish model (51, 52, 62, 94). An interesting outcome of enchained growth is that the bacteria within an agglutinated aggregate are monoclonal, as opposed to a heterogeneous mixture of bacteria found in classically agglutinated aggregates (62). Thus, when the agglutinated bacteria are expelled from the host, enchained growth could ultimately lead to the extinction of particular bacterial clones from the metagenome, thereby reducing the genetic diversity of a specific bacterial strain or species (62, 123). Although antibody-agglutination does not need to specifically target microbes utilizing flagellar motility, agglutination renders flagellated bacteria non-motile and thereby susceptible to clearance from the host.

#### 3.2.3 Immobilization

While agglutination may be an effective strategy when concentrations of bacteria are high or when they are actively dividing, what strategies might be better suited for single flagellated microbes swimming through mucus towards the epithelium? Schroeder and colleagues found that antibody imbedded in mucus can immobilize microbes, independent of agglutination and neutralization (96, 97) (**Figure 2D**). This ability depends on a synergy between antibody and mucus. The Fc domain of IgG has weak interactions with mucins, which are dependent on antibody glycosylation. In isolation, these weak interactions are unable to prevent antibody diffusion within the mucus (96, 97); however, like Velcro, a cumulative force from multiple weak interactions occurring between IgG-coated bacteria and mucus leads to the bacterial immobilization (**Figure 3D**) (96–98). Through this mechanism, antibody can restrict mobility irrespective of bacterial concentration or division. 

## 4 Concluding Remarks

From a microbe’s perspective, current literature supports that flagella can provide microbes a fitness advantage in the host, because of the ability to localize to beneficial sites. Luminal bacteria can use flagella-driven chemotaxis to locate nutrient micro-niches produced by inflammation or epithelial damage, which they can exploit for a growth advantage over competitors. Alternatively, some bacteria utilize motility to access the epithelium, permitting lifestyles that require adherence, invasion, and/or translocation. From the host’s perspective, flagellar motility can represent varying degrees of risk, which over an evolutionary timescale resulted in the development of numerous and often redundant defense mechanisms that collectively work to inhibit flagellar motility and/or eliminate flagellated microbes.

When a host is exposed to a novel flagellated microbe, the innate immune system can combine motility-inhibiting peptides and proteins, along with increased mucus secretion, to limit access to the epithelium and promote the elimination of immobilized microbes. While these initial barriers are not impermeable, the collective effects of innate responses reduce microbe-epithelium interactions and limit potential damage to the mucosa. When required, additional immune resources are recruited to eliminate the threat. An antibody response can provide long term fortification of the mucus through motility inhibition. In this case, upon subsequent re-challenge by the antibody-targeted microbe, its flagellar motility will be met with a combined response of both the innate and adaptive immune system. Interestingly, many of the mechanisms of motility inhibition are non-lethal, which may reflect a strategy aimed to avoid collateral damage to the cohabitating microbiota. Additionally, the host’s active “pruning” or attenuation of commensal flagella expression could represent a co-evolved strategy where microbes benefiting from membership in the commensal microbiota need to follow the “rules” set by the host and not employ flagella.

Although innate effectors and antibody may target different epitopes and with different affinities, there are redundancies in their mechanisms of motility inhibition. For example, agglutination is a method employed by both antibody and innate effector molecules such as HD6. Likewise, the agglutination-independent immobilization of microbes occurs with both antibody and innate effectors such as LYPD8. While antibody accomplishes this through cumulative antibody-mucus interactions, the mechanism employed by LYPD8 remains to be elucidated. Thus, our understanding of this mechanism of antibody-mediated motility inhibition could provide clues on how LYPD8, or other innate molecules are able to inhibit motility.

Technological innovations in imaging and sequencing are advancing our understanding of the roles of microbial motility within the host, as well as the motility-directed countermeasures of the host. Recent advances in sequential fluorescence in situ hybridization have enabled researchers to observe the differential expression of hundreds of genes at single-bacterium resolution (124). This technique could allow future studies to broaden the scope of our understanding, for example, from one based largely on model enteric pathogens, to include how flagellated members of the commensal microbiota also use motility within the host. Likewise, further investigations of the host mucosal immune system will likely uncover novel effector molecules, synergies, and molecular mechanisms that inhibit microbial motility. The intersection between motile microbe and host remains a complex and nuanced subject, with many of its intricacies still unknown.

## Author Contributions

Both authors contributed to the conceptual framework and writing of the review. All authors contributed to the article and approved the submitted version.

## Funding

National Institutes of Health T32AI06055, National Institutes of Health R37AI32738, and National Institutes of Health U01AI125926 (Mucosal Immunology Study Team).

## Conflict of Interest

The authors declare that the research was conducted in the absence of any commercial or financial relationships that could be construed as a potential conflict of interest.

## Publisher’s Note

All claims expressed in this article are solely those of the authors and do not necessarily represent those of their affiliated organizations, or those of the publisher, the editors and the reviewers. Any product that may be evaluated in this article, or claim that may be made by its manufacturer, is not guaranteed or endorsed by the publisher.
